# Retrospective Study of *Listeria monocytogenes* Isolated in the Territory of Inner Eurasia from 1947 to 1999

**DOI:** 10.3390/pathogens8040184

**Published:** 2019-10-11

**Authors:** Ekaterina K. Psareva, Irina Yu. Egorova, Elena A. Liskova, Irina V. Razheva, Nadezda A. Gladkova, Elena V. Sokolova, Eugene A. Potemkin, Pavel A. Zhurilov, Tatyana V. Mikhaleva, Andrei A. Blokhin, Yaroslava M. Chalenko, Denis V. Kolbasov, Svetlana A. Ermolaeva

**Affiliations:** 1 Federal Research Center for Virology and Microbiology, Nizhny Novgorod Research Veterinary Institute Branch, Nizhny Novgorod 603950, Russiarazheva64@bk.ru (I.V.R.); nivigladkova@yandex.ru (N.A.G.); sokol.e1ena@yandex.ru (E.V.S.); jeka89290462295@gmail.com (E.A.P.); Zhurilov95@bk.ru (P.A.Z.); and.bloxin2010@yandex.ru (A.A.B.); yaroslavazaka@yandex.ru (Y.M.C.); drermolaeva@mail.ru (S.A.E.); 2 Federal Research Center for Virology and Microbiology, Volginsky 601125, Russia; iegorova@list.ru (I.Y.E.); kolbasovdenis@gmail.com (D.V.K.); 3 Federal Research Center for Virology and Microbiology, Samara Research Veterinary Institute Branch, Samara 443013, Russia; Tatyanamihaleva@mail.ru; 4 Gamaleya Research Centre of Epidemiology and Microbiology, Moscow 123098, Russia

**Keywords:** *Listeria monocytogenes*, listeriosis, food infections, virulence, multilocus sequence typing, clonal complex, phylogenetic lineages

## Abstract

Listeriosis is one of the most significant humans and animals foodborne infectious diseases. Here, we characterized 48 *Listeria monocytogenes* strains isolated in the territory of inner Eurasia during the second half of the 20th century. A total of 23 strains (52.3%) were susceptible to the nine antibiotics tested, 30.43%, 15.22%, and 8.7% were resistant penicillin G, ampicillin, and enrofloxacin, respectively. We applied the multilocus sequence typing (MLST) scheme to determine the phylogenetic positions of the strains. All but one strain belonged to the II phylogenetic lineage, and the majority of the strains belonged to one of the previously described clonal complexes (CCs). More than 60% of the strains belonged to the clonal complex CC7 that prevailed among all sources, including cattle (58%), small ruminants (64%), rodents (71%), and humans (50%). Further, CC7, CC101, and CC124 were found among human isolates. The MLST scheme was supplemented with virulence gene analysis. In total, eight *inlA*, six *inlB*, and six *inlC* allelic variants were found, and all but one strain carried one of the two *inlE* alleles. Most strains (62.5%) belonged to the same multivirulence locus sequence typing (MvLST) type, which includes CC7, *inlA* allele 4, *inlB* allele 14, *inlC* allele 6, and *inlE* allele 8.

## 1. Introduction

Listeriosis is an infectious disease of humans and animals that results in high hospitalization and fatality rates [1,2]. The annual incidence of listeriosis ranges from 0.2 to 0.5 cases per 100,000 population, while average fatality rates are estimated to be 20%–30% of hospitalized patients and can reach 40% in outbreaks, including elderly persons or immunosuppressed patients [3,4]. 

Listeriosis is currently considered to be one of the most significant food infections [5,6]. The worldwide epidemic situation continues to deteriorate for a number of reasons, including specific biological features of the causative agent of listeriosis, the Gram-positive bacterium *Listeria monocytogenes* [7]. *L. monocytogenes* is widely spread in the environment [8,9,10,11]. It is able to survive in saprophytic conditions, to effectively withstand bacterivorous organisms inhabiting terrestrial and aquatic ecosystems, and to cause disease in a wide range of domestic and wild animals [7,9,12,13,14,15,16,17]. The wide distribution of *L. monocytogenes* in the soil contributes to animal reinfection and pathogen conservation in nature [15,18]. Ruminants fed on silage are at a higher risk of contracting *L. monocytogenes* infection. In small ruminants, *L. monocytogenes* often causes neurological disease and abortion [19,20]. Adult swine and cows can be infected by *L. monocytogenes* but rarely develop neurological disease [2,21,22]. Meanwhile, *L. monocytogenes* is a common contaminant of raw milk, and its occurrence in the bulk tank milk of the dairy cow ranges from 2% to 7% [23,24,25]. In general, wild animals are asymptomatic carriers that shed the bacterium in their feces, although invasive infection has been described in wild small rodents and both invasive infection and fatal cases have been described in wild deer [9,17,26,27,28]. 

The species *L. monocytogenes* has a clonal structure and is divided into four evolutionary lineages (I, II, III, and IV) occupying different, but overlapping, ecological niches [29,30,31]. Most *L. monocytogenes* isolates belong to lineages I and II. The majority of human listeriosis outbreaks and many sporadic cases are associated with lineage I isolates [32]. In bovine, lineage I is predominantly isolated from cases of encephalitis [19,20]. Lineage II strains, which are common in foods, seem to be widespread in natural and farm environments and are also commonly isolated from sporadic human clinical cases and animal listeriosis cases, which are equally represented by isolates from cases of encephalitis versus septicemia and fetal infection [31,32,33]. Lineage III and IV strains are rare and are predominantly isolated from animal sources [34].

The phylogenetic structure of the *L. monocytogenes* species was investigated using multiple molecular methods, such as multilocus enzyme electrophoresis and whole genome sequencing [35,36,37]. The method of multilocus sequence typing (MLST), based on sequencing of seven housekeeping genes, is a powerful technique that provides high resolution and specificity in addition to unambiguous results and rigor in analysis [28,33,38]. Introduction of the concept of clonal complexes (CCs) as a group of one-marker deviations provided the means for large-scale analysis of clonal distribution worldwide [17,34,38,39].

In this work, we used the MLST scheme [28] to typify *L. monocytogenes* strains that were isolated from humans and domestic animals in the territory of inner Eurasia—a huge geographic region covering Eastern Europe, Central Asia, and Russia [39]—during the second half of the 20th century. The strains are kept in the National Collection of the Federal Research Center for Virology and Microbiology (Volginsky, Russia). The MLST scheme was supplemented with analysis of the virulence genes encoding proteins of the internalin family. Such a multivirulence locus sequence typing (MvLST) scheme has been used to characterize current *L. monocytogenes* isolates obtained in the territory of Russia from natural and anthropogenic environments [16,40]. This retrospective MvLST analysis allowed for characterization of the strains isolated in this territory when the area was isolated from other parts of the world, and allowing for comparison with modern data.

## 2. Results

### 2.1. Antibiotic Resistance

All 48 strains tested in this study were sensitive to kanamycin, streptomycin, and neomycin. Natural resistance to polymyxin B was observed in 44 strains. Total of 23 strains (52.3%) were susceptible to all nine tested antibiotics besides polymyxin B. Among strains with resistance, 20 strains (45.5%) were resistant to one antibiotic and five strains (11.4%) were resistant to two antibiotics. The highest percentage of observed resistance was to penicillin G (30.43%), followed by ampicillin (15.22%), and enrofloxacin (8.7%) (Table 1).

### 2.2. L. monocytogenes Virulence 

When tested in guinea pigs, 40 *L. monocytogenes* strains (83%) caused progressive conjunctivitis and keratitis with generalized infection. In one (2%) case, the generalized infection ended in death (strain 211). Seven strains (15%) were incapable of causing conjunctivitis and keratitis.

A total of 17 strains were additionally tested in mice. Strains were selected by random sampling from various sources. Fourteen strains (82%) were virulent for mice, for which the LD_50_ ranged from 10^4^ to 10^7^ CFU per mouse (Table 2). Two strains (11.8%), including strain 2598 isolated from rabbit and strain 39 isolated from guinea pig, were moderately virulent for mice, although they caused purulent keratoconjunctivitis in guinea pigs. Strain “A,” isolated from ticks, was avirulent for mice and unable to cause keratoconjunctivitis in guinea pigs. 

### 2.3. MLST Typing of L. monocytogenes Strains

More than half (60.4%) of all strains (29 of 48) belonged to the clonal complex CC7. Twelve of them (41.4%) belonged to the parental CC7 sequence type ST7. Other CC7 strains belonged to six established and nine new described sequence types (Table 3). Sequence types ST12, ST177, and ST23 were each identified in two strains. Eleven other sequence types (ST85, ST98, ST106, ST519, ST124, ST481, ST757, ST101, ST21, ST252, ST308, and ST2) were represented by a single strain. All but one *L. monocytogenes* strain (98%) belonged to the II phylogenetic lineage, and only strain K-23 (ST252; CC1) belonged to the I phylogenetic lineage. Nine previously described CCs [32] were found among the *L. monocytogenes* strains (Table 3).

Besides CC7, four more clonal complexes were represented by more than one sequence type. The clonal complex CC18 was represented by three sequence types, and CC124, CC101, and CC177 were represented by two sequence types. At least one sequence type found in strain 45 from sheep detected in Kazakhstan did belong to new ST1549 and clonal complex CC307. In total, eight new sequence types were described. The studied strains information is presented at the L. monocytogenes MLST database at https://bigsdb.pasteur.fr/.

### 2.4. Characterization of CC Distribution among Sources of Isolation

**Figure 1:**
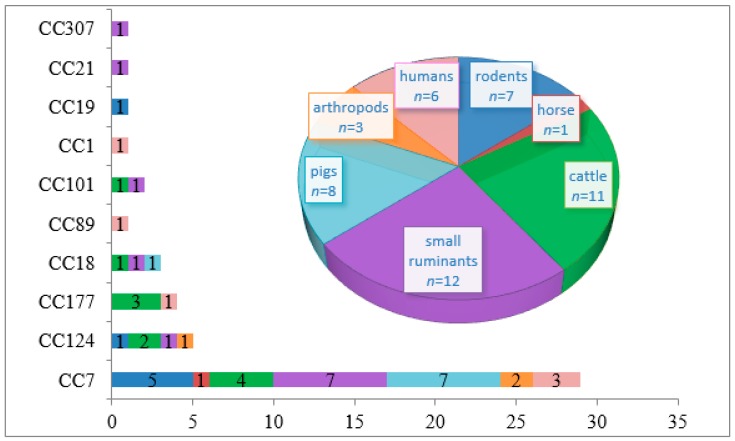
The clonal complexes (CCs) and sources of *L. monocytogenes* isolates, including isolates from small ruminants (*n* = 12; 25%), cattle (*n* = 11; 23%), humans (*n* = 6; 12.5%), pigs (*n* = 8; 16.7%), rodents (*n* = 7; 14.6%), arthropods (*n* = 3; 6.25%), and a horse (*n* = 1; 2%).

The 48 tested *L. monocytogenes* strains included isolates from small ruminants (25%), cattle (23%), humans (12.5%), pigs (16.7%), rodents (14.6%), arthropods (6.25%), and a horse (2%) (Figure 1 and Table 1). Strains belonging to the clonal complex CC7 prevailed among all sources, including cattle (7 of 12; 58%), small ruminants (7 of 11; 64%), rodents (5 of 7; 71%), humans (3 of 6; 50%), arthropods (2 of 3; 66.7%), and horse (1 of 1; 100%). 

With the exception of CC7 strains, strains isolated from small ruminants, cattle, and humans were quite heterogeneous, although all but one belonged to the II phylogenetic lineage. Five isolates from small ruminants belonged to CC124, CC18, CC101, CC21, and a new ST which has not been previously described. CC177 (*n* = 3), CC124 (*n* = 2), CC18 (*n* = 1), and CC21 (*n* = 1) were found in strains isolated from cattle. Besides isolates from cattle and small ruminants, CC124 was also found in strains isolated from a rabbit and ticks. New STs profiles belonging to CC18 and CC19 were found in strains isolated from pig and rabbit, respectively (Figure 1). Strains isolated from humans belonged to CC7 (*n* = 3), CC89 (*n* = 1), CC177 (*n* = 1), and CC1 (*n* = 1, I phylogenetic lineage). 

### 2.5. Distribution of the L. monocytogenes Clonal Complexes on the Territory of Inner Eurasia

**Figure 2:**
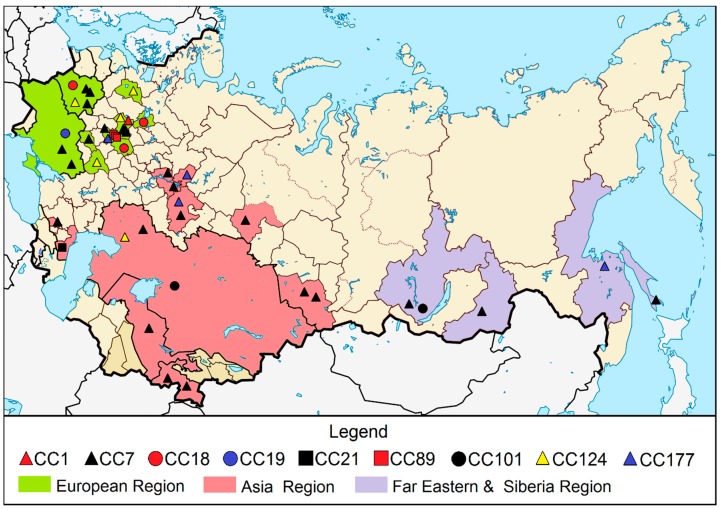
Map showing locations in inner Eurasia where *L. monocytogenes* strains were isolated.

All *L. monocytogenes* strains were obtained from inner Eurasia territories. To simplify analysis, the regions were divided into (1) the European region, which unites the territories of current eastern European countries (Ukraine and Belarus) and central regions of Russia (Moscow, Voronezh, Kursk, Ryazan, Yaroslavl, Tula, and Novgorod regions); (2) the Asia region, which includes territories of Central Asian countries (Tajikistan, Kazakhstan, and Uzbekistan) and territories of the Russian Federation (North Caucasus, Dagestan, Ural, Volga, and Altai regions); and (3) the far eastern and Siberia region of the Russian Federation (Irkutsk, Sakhalin, Khabarovsk, and Chita regions) (Figure 2).

All but one *L. monocytogenes* strain isolated in the European region belonged to the II phylogenetic lineage and included the main clonal complexes CC7 (*n* = 15), CC124 (*n* = 4), CC177 (*n* = 1), CC18 (*n* = 4), CC89 (*n* = 1), and CC19 (*n* = 1). Only one strain, isolated in Moscow in 1988, belonged to the I phylogenetic lineage. Strains isolated in the Asia region belonged to CC7 (*n* = 11), CC177 (*n* = 2), CC124 (*n* = 1) CC21 (*n* = 1), and CC101 (*n* = 1), and one strain belonged to a new sequence type that was not associated with any described clonal complex. In the far eastern and Siberia region, strains of CC7 (*n* = 3), CC177 (*n* = 1), and CC101 (*n* = 1) were found. Therefore, CC7 was the most frequent in the whole territory in inner Eurasia.

### 2.6. Internalin Gene Diversity in L. monocytogenes Isolates

In this study, previously described internal fragments of the internalin genes *inlA*, *inlB*, *inlC*, and *inlE* [39] were sequenced to supplement the MLST analysis with virulence gene analysis.

In total, 8 *inlA* allelic variants were found. Alleles 1, 4, 6, 8, 9, and 12 have been described in previous studies while alleles 14 and 15 were detected for the first time. The *inlA* gene was not detected in strain 45 isolated from sheep in Kazakhstan, which belongs to a newly described ST. It is noteworthy that each allele of the *inlA* gene correlated with a specific CC (Table 4).

All studied *L. monocytogenes* strains contained one of six alleles of the *inlB* gene, and the dominant allele 14 was detected in 35 (73%) strains belonging to CC7, CC177, CC21, and CC19. Strains belonging to CC1, CC124, CC101, and CC89 carried alleles 1, 12, 15, and 17, respectively (Table 4). Strain 45, which belongs to a new sequence type, did not correlate with any clonal complex carrying *inlB* allele 16 (data not shown).

The variability of *inlC*, and especially *inlE*, was low. In total, 6 *inlC* alleles were found, among which allele 6 was predominant and found in 40 of 48 (83%) strains belonging to CC7, CC21, CC19, CC124, CC177, and CC18. Other *inlC* alleles were distributed in a CC-specific manner and were represented in single strains. As for *inlE*, all but one strain carried one of two alleles, either 6 or 8. The only exception was the phylogenetic lineage I strain K-23, which carried alleles distinct from the other studied genes. Interestingly, all strains carrying *inlE* allele 8 were isolated in the Asian part of inner Eurasia, and all but one strain carrying allele 6 of *inlE* was isolated in the European territory. The exception was the strains of CC7 that carried allele 8 and were widely spread over the whole inner Eurasia territory. 

Polymorphism among housekeeping genes included in the MLST scheme and virulence genes included in the internalin profile (IP) was compared only for strains of lineage II, to avoid the introduction of false variability which would have been caused by the addition of a single lineage I strain into the analysis because of a high percentage of conservative substitutions between lineages [16,38]. It was shown that for the studied collection, the polymorphism of virulence genes was lower or comparable with the polymorphism of housekeeping genes. Particularly, the virulence gene *inlE* had the lowest number of alleles relative to all studied genes, and the housekeeping gene *ldh* had the highest number of alleles (2 vs. 18) (Table 5).

**Figure 3:**
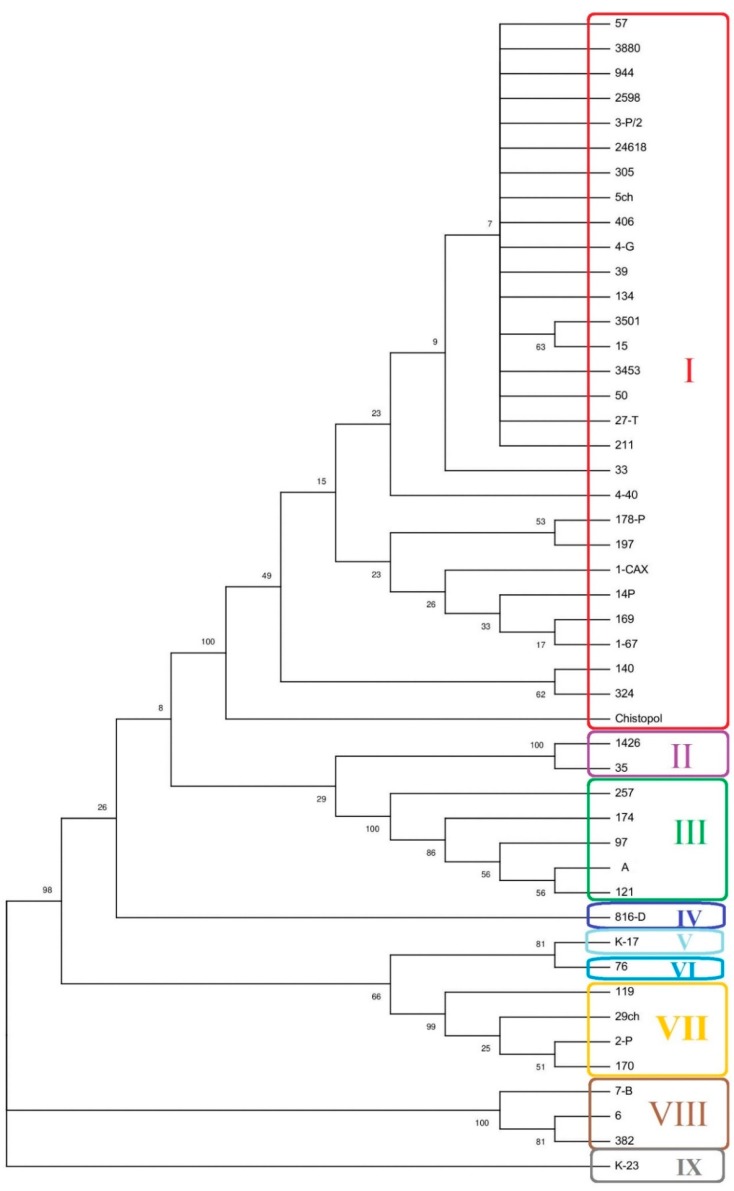
The dendrogram was constructed based on the MLST sequence types and internalin gene alleles.

A phylogenetic tree was constructed on the basis of concatenated sequences of the MLST and internalin gene alleles (Table 4 and Figure 3). The major cluster, Cluster I, was identified from phylogenetic analysis, and included strains containing CC7, *inlA* allele 4, *inlB* allele 14, *inlC* allele 6, and *inlE* allele 8. Other clusters (II–IX) were observed and clustered according to clonal complexes of *L. monocytogenes* strains and internalin gene alleles profiles (IP). Cluster II included two strains from sheep and cow belonging to CC101 with the internalin profile (IP) 14, 20, 15, 8 for *inlA*, *inlB*, *inlC*, and *inlE*, respectively. Cluster III was represented by five strains isolated from different sources in Europe and belonged to CC124 and IP 6, 12, 6, 6. Clusters IV–VI were represented by one strain each belonging to distinct clonal complexes (CC21, CC19, CC89, respectively), and carrying distinct *inlA* alleles (alleles 12, 9, and 15, respectively). These three clusters had the same alleles of *inlB* and *inlE* (14 and 8, respectively). Clusters IV and V shared the *inlC* allele (6) while Cluster VI carried the *inlC* allele 17. Four strains from the cattle had Cluster VII profile and belonged to CC177 with IP 8, 14, 6, 8 that differed from IP of Clusters IV and V only by the *inlA* allele. Cluster VIII was represented by three strains obtained from different sources in Europe and belonged to CC18 with IP 12, 13, 6, 6. The only one strain isolated from the human case belonged to the 1 phylogenetic lineage, Cluster IX, CC1 and IP 1,9,1,3. 

## 3. Discussion

Here, we performed a retrospective study of *L. monocytogenes* strains isolated in the territory of inner Eurasia in the second part of the 20th century, when countries of the former Soviet Union were to certain extent isolated from the rest of the world. Animal isolates prevailed in the studied collection while only six human isolates were included that is in line with the rare occurrence of human listeriosis until the 1980s [41,42,43]. Food isolates were not included into the study as food products began to be considered as a source of listeriosis in the 80s, and a mandatory check of products for listeriosis was introduced in Russia in 2002, so food isolates dated before 1990 are absent from the National Collection of the Federal Center for Virology and Microbiology (Volginsky, Russia), which was the strain source. 

The analysis of antibiotic resistance showed that about half of the strains were sensitive to all antibiotics tested. This is consistent with the rarer use of antibiotics during that period, and is in stark contrast with the modern prevalence of antibiotic-resistant strains among animal and environmental *L. monocytogenes* isolates [44,45,46].

The obtained results suggested the prevalence of lineage II strains among animal isolates obtained in the territory of inner Eurasia from 1947 to 1999. These data are in line with previously obtained results which demonstrated the prevalence of lineage II *L. monocytogenes* strains among wild animals inhabiting natural foci of infection in the European part of Russia [16]. In general, the comprehensive analysis of isolates obtained in European countries showed the prevalence of lineage I among clinical isolates, and lineage II among food and environmental isolates [20,44,47]. Both lineage I and II strains were isolated from wild animals that inhabited different regions of Germany and Austria [48]. Prevailing of Lineage II was described in some studies performed in Sweden [49,50], although comparison with these studies was restricted by inconsistence of isolation sources. A more consistent study from the point of view of the isolation source study showed longitude prevalence of Lineage II strains at three Finnish dairy farms during 2013 to 2016 [15]. 

The current study included only five strains isolated in the far eastern part of Eurasia, all of which belong to lineage II. The phylogenetic lineage I prevailed among isolates obtained from invasive diseases in humans and wild animals in the far eastern region of Russia at the beginning of the 21st century [9]. Other studies demonstrated similar representations of the I and II phylogenetic lineages among *L. monocytogenes* strains isolated from the intestinal contents and feces of wild animals in Japan and China in this century [25,51]. Taken together, the available data suggest that the distribution of the I and II lineages might be different for the European and Central Asian parts of Eurasia and its far eastern part. More representative studies are required to clarify the phylogenetic pattern of *L. monocytogenes* circulating in this region.

Further, recent as well as earlier studies have demonstrated the prevalence of the clonal complex CC7 among isolates obtained from the European and Central Asian parts of inner Eurasia (see Table 4 and Figure 1, [16,38]). CC7 is one of the largest-spreading clonal complexes worldwide. It includes the epidemic clone VII (ECVII), which is associated with the foodborne outbreak of listeriosis that was associated with the consumption of cantaloupe during 2011 in the United States [52]. The distribution of *L. monocytogenes* clones varies across countries around the world. Clonal complexes CC1 and CC2 of phylogenetic lineage I have prevailed in all regions of the world, with the exception of North Africa for CC1. CC2 clones have been found in 30 countries. CC3 is one of the most common clones in all regions, while CC9 of the lineage II was shown to be the third largest in Europe and the Western Hemisphere [31]. The predominant clonal complexes in China are CC9 and CC8 in food and ST87 in clinics [53,54]. In the last decade, CC6 noticeably increased in significance as a causative agent of human listeriosis in Africa, Europe, and North America [31,33,55,56,57]. In Russia, current human listeriosis is associated with the CC1, CC2, and CC20 strains [38].

Analysis of virulence gene variability revealed a difference between the distribution of *inlA* and *inlB* respectively encoding two major invasion factors, InlA and InlB. While *inlA* is distributed according to a phylogenetic position and is well correlated with clonal complexes, the *inlB* allele 14 was found in strains belonging to four of the nine revealed clonal complexes. This prevalence might be due to some selective advantage of this allele. Recently, we demonstrated that *inlB* allele 14 provided the highest level of *L. monocytogenes* dissemination to the internal organs of mice when infection was performed via intragastric inoculation, which is the best model of the natural infection process [58]. All lineage II strains isolated from internal mouse organs in this and previous studies carried the *inlB* allele 14, and mice might be an important host for *L. monocytogenes* in the natural focus of infection [38]. Variability analysis of two other IP genes, *inlC* and especially *inlE*, showed that their sequences were as conserved as the most conservative housekeeping genes. 

Taken together, the obtained results demonstrate that *L. monocytogenes* strains belonging to CC7 prevailed among isolates obtained in the territory of inner Eurasia in the second half of the 20th century. Resistance to antibiotics occurred among these strains, but about 50% of the isolates were sensitive to all tested antibiotics with the exception of polymyxin B. Analysis of virulence gene variability demonstrated different patterns of distribution for genes encoding proteins of the internalin family among these strains.

## 4. Materials and Methods

### 4.1. Bacterial Strains and Culturing Conditions

A total of 48 *L. monocytogenes* strains were obtained from the National Collection of the Federal Research Center for Virology and Microbiology (Volginsky, Russia) for further analysis (Table 6). Independent isolates were chosen on the basis of having been isolated from humans and animals in different regions of inner Eurasia from 1947 to 1999. Strains isolated from animal insect and human parasites were included. All strains from biological sources (with the exception of rodents and insects) were isolated from postmortal material taken from animals who died from listeriosis, daveric (sectional) material. Isolates were obtained from animals which died from listeriosis. Serotypes were characterized with antisera. Strains were kept lyophilized at −18 °C with bovine serum or gelatin agar medium as a stabilizer.

Lyophilized *L. monocytogenes* cultures were restored using 1 mL of tryptone soya yeast extract broth (TSB-YE, HiMedia, India). The strain suspension was immediately plated on tryptone soya yeast extract agar (TSA-YE, HiMedia, India). Plates were incubated at 37 °C for 24 h; then, colonies were counted and up to 10 typical colonies were replated for further identification and characterization. The remainder of the initial suspension was supplemented with sterile TSA-YE and incubated at 37 °C for 24 h without shaking to guarantee culture viability in the absence of colony growth on agar plates.

All cultures were confirmed as *L. monocytogenes* using Microgen Listeria ID (Microgen Bioproducts, U.K.). Additionally, the colonies from TSA-YE were verified by Gram staining, catalase reactions, oxidase tests, and motility at 20–25 °C (Listeria Motility Medium, HiMedia, India).

### 4.2. Antibiotic Resistance Test

*L. monocytogenes* strains were tested for their resistance to antibiotics using the disc diffusion method on Mueller–Hinton agar (HiMedia, India), as described by the performance standards for antimicrobial disk and dilution susceptibility test for bacteria isolated from animals (CLSI) [59]. Penicillin G (10 µg/disc), enrofloxacin (5 µg/disc), ampicillin (10 µg/disc), tetracycline (30 µg/disc), levomycetin (chloramphenicol) (30 µg/disc), kanamycin (30 µg/disc), tylosin (15 µg/disc), streptomycin (30 µg/disc), polymyxin B (30 µg/disc), and neomycin (30 µg/disc) were used (NICF, LLC; St. Petersburg, Russia). These antibiotics were chosen for use or preference in veterinary medicine for the treatment of infectious diseases in animals in Russia. After 24 h of incubation, the zones of inhibition were measured (mm), and the strains were categorized as susceptible, intermediate, or resistant to specific antibiotics as per the criteria of CLSI [59]. The breakpoints of *Staphylococcus*, *Streptococcus*, and *Enterococcus* species resistance were considered since no resistance criteria exist in the CLSI guidelines for *Listeria* susceptibility testing [25]. 

### 4.3. Anton’s Eye Test

Anton’s eye test was performed on guinea pigs [60]. Strains were recognized as pathogenic of purulent keratoconjunctivitis when it developed in guinea pigs within 3–5 days.

### 4.4. L. monocytogenes Virulence in Mice

Virulence was estimated by the 50% lethal dose (LD_50_), determined by the probit method, using white laboratory outbred Swiss mice weighing 14–16 g strains were randomly selected among isolates obtained from a particular source. Bacteria were grown on TSA-YE slopes (HiMedia, India) for 18 h at 37 °C. The culture was standardized turbidimetrically and diluted appropriately in phosphate-buffered saline (PBS, pH 7.3). For each strain, five out of six mice were injected intraperitoneally with 0.5 mL of dilution. Dilutions were 1.0 × 10^9^, 2.0 × 10^8^, 4.0 × 10^7^, 8.0 × 10^6^, and 1.6 × 10^6^ CFU per animal. Each inoculum was checked by determining the viable counts on TSA-YE plates. 

The LD_50_ was calculated using the following formula:

lg LD_50_ = DN − lgδ(∑ Li − 0.5)

where lgDN is the logarithm of the maximum infecting dose, lgδ is the logarithm of the multiplicity of dilution (the ratio of each subsequent dose to the previous one), Li is the ratio of the number of animals who died from a given dose of the inoculation to the total number of animals inoculated in this dose, and ∑ Li is the sum of the Li values calculated for all doses tested.

All strains with LD_50_s from 1.0 × 10^4^ to 1.6 × 10^7^ CFU per mouse were considered as virulent. A strain was nonvirulent if it did not cause death in the injected group of mice 7 days after injection with 1.0 × 10^9^ CFU [44]. Animal experiments were performed according to the European Convention for the Protection of Vertebrate Animals used for Experimental and Other Scientific Purposes (Strasbourg, 1986). All procedures involving animals were approved by the Ethic Committee of the Federal Research Center for Virology and Microbiology (Volginsky, Russia) (ref. no. 5 on February 28, 2019). After infection, the surviving mice were euthanized by CO_2_ asphyxiation.

### 4.5. PCR 

Lysates of overnight *L. monocytogenes* cultures were obtained as previously described [38]. The Encyclo Plus PCR kit (Evrogen, Russia) and primers (Syntol, Russia) were used for DNA amplification. The MLST scheme based on the sequences of seven housekeeping genes that was developed by Ragon et al. was used [28]. The housekeeping gene fragments were amplified as previously described, with modifications [16]. The following temperature conditions were used: 94 °C, 2 min; (92 °C, 30 s; 55 °C, 30 s; 72 °C, 2 min) × 30; 72 °C, 10 min; melting temperatures (Tm) were 60 °C for *bglA* and *ldh.* PCR products were evaluated by electrophoresis in 1% agarose gel. Amplified DNA was eluted from the reaction mixture using Cleanup Standard (Evrogen, Russia).

PCR amplification of the internal fragments of four internalin genes (*inlA*, *inlB*, *inlC*, and *inlE*) was performed as previously described [16,38]. 

### 4.6. PCR Product Sequencing

PCR products were sequenced on both strands according to the BigDye Terminator 3.1 Cycle Sequencing protocol for the Genetic Analyzer 3130 of Applied Biosystems/Hitachi. Electrophoretic DNA separation was performed in 50-cm capillaries with POP7 polymer.

### 4.7. Sequence Analysis

Sequences were proofread and assembled in Chromas Lite MFC Application version 2.1.1.0. DNA alignment was performed using ClustalX2. Dendrograms were constructed with Mega X version 10.1 (https://www.megasoftware.net/) [61]. Allelic numbers and profiles (genotypes, sequence types (STs)) were determined using the *L. monocytogenes* MLST database (https://bigsdb.pasteur.fr/listeria/listeria.html). Nucleotide diversity was analyzed with DnaSP version 6 [62].

## Figures and Tables

**Table 1:** Antibiotic resistance of *L. monocytogenes* strains.

Antibiotic	Number of Resistant Strains
Penicillin G	14
Enrofloxacin	4
Ampicillin	7
Tetracycline	1
Chloramphenicol	1
Kanamycin	0
Tylosin	2
Streptomycin	0
Polymyxin B	44
Neomycin	0

**Table 2:** Virulence of *L. monocytogenes* strains for Swiss mice.

LD_50_	Number of Strains in a Particular Source								
	Humans	Cattle	Goat	Pig	Mouse	Rat	Sheep	Rabbit	Tick
10^4^–10^7^	1	3	1	2	2	1	2	2	0
10^7^–10^9^	0	1	0	0	0	0	0	1	0
≥10^9^	0	0	0	0	0	0	0	0	1

**Table 3:** Phylogenetic characterization of the *L. monocytogenes* strains included in the study.

Phylogenetic Lineage	Clonal Complex (CC)	Sequence Type (ST)	Number of Strains in a Particular Source						
			Humans	Cattle	Small Ruminants	Pigs	Rodents	Horse	Arthropods
II	CC7	ST7	1	0	3	3	5	0	0
		ST12	1	1	0	0	0	0	0
		ST23	0	1	0	1	0	0	0
		ST85	0	0	1	0	0	0	0
		ST98	0	0	0	0	0	0	1
		ST106	0	0	0	0	0	1	0
		ST519	0	0	0	1	0	0	0
		**ST1534**	0	0	0	0	0	0	1
		**ST1535**	0	0	0	1	0	0	0
		**ST1536**	0	1	0	0	0	0	0
		**ST1537**	0	0	1	0	0	0	0
		**ST1538**	0	1	0	0	0	0	0
		**ST1539**	1	0	0	0	0	0	0
		**ST1540**	0	0	1	0	0	0	0
		**ST1541**	0	0	0	1	0	0	0
II	CC124	ST124	0	1	0	0	0	0	1
		**ST1550**	0	0	0	0	1	0	0
		**ST1551**	0	1	0	0	0	0	0
		**ST1552**	0	0	1	0	0	0	0
II	CC177	ST177	0	2	0	0	0	0	0
		**ST1542**	0	1	0	0	0	0	0
		**ST1543**	1	0	0	0	0	0	0
II	CC18	ST481	0	0	1	0	0	0	0
		**ST1544**	0	0	0	1	0	0	0
		**ST1545**	0	1	0	0	0	0	0
II	CC89	**ST1547**	1	0	0	0	0	0	0
II	CC101	ST101	0	0	1	0	0	0	0
		**ST1548**	0	1	0	0	0	0	0
II	CC19	**ST1546**	0	0	0	0	1	0	0
II	CC21	ST21	0	0	1	0	0	0	0
II	CC307	**ST1549**	0	0	1	0	0	0	0
I	CC1	ST252	1	0	0	0	0	0	0

**Table 4:** Clonal complexes and internalin gene alleles of *L. monocytogenes* strains.

	Clonal Complexes (Number of Strains)	Gene Allele			
		*inlA*	*inlB*	*inlC*	*inlE*
I	CC7 (29)	4	14	6	8
II	CC101 (2)	14	20	15	8
III	CC124 (5)	6	12	6	6
IV	CC21 (1)	12	14	6	8
V	CC19 (1)	9	14	6	6
VI	CC89 (1)	15	15	17	6
VII	CC177 (4)	8	14	6	8
VIII	CC18 (3)	12	13	6	6
IX	CC1 (1)	1	9	1	3

**Table 5:** Polymorphism of gene fragments included in the multivirulence locus sequence typing (MvLST) (multilocus sequence typing (MLST) + internalin profile (IP)) scheme for the strains of the II phylogenetic lineage.

Gene	Length	N Alleles	Mutations		π ^a^	InDel ^b^	Rm ^c^
			Synon	Nonsynon			
**MLST**							
*abcZ*	537	4	8	1	0.00838	0	0
*bglA*	399	7	11	0	0.00979	0	1
*cat*	486	8	8	3	0.00845	0	1
*dapE*	462	9	27	6	0.01852	0	3
*dat*	471	4	4	0	0.00425	0	0
*lhkA*	480	3	1	1	0.00278	0	0
*ldh*	459	18	5	6	0.00768	6	4
**IP ^d^**							
*inlA*	648	7	13	5	0.01190	0	3
*inlB*	618	5	5	6	0.00906	0	2
*inlC*	587	5	10	5	0.01126	0	0
*inlE*	558	2	1	1	0.00358	0	0

^a^ π—nucleotide diversity; ^b^ InDel—the number of inserted/deleted nuleotides; ^c^ Rm—minimum number of recombination events; ^d^ IP—internalin profile.

**Table 6:** *Listeria monocytogenes* strains used in the study.

No.	Strain	Year	Source	Location	Serovar	ST	CC
1	4-40	1947	horse	Moscow	1/2a	ST106	CC7
2	134	1952	vole mouse	Moscow region	1/2a	ST7	CC7
3	15	1952	pig	Belarus	1/2a	ST519	CC7
4	6	1952	pig	Ryazan oblast	1/2a	**ST1544**	CC18
5	A	1952	ticks	Kazakhstan	1/2a	ST124	CC124
6	382	1954	cow	Yaroslav region	1/2a	**ST1545**	CC18
7	197	1955	ticks	Ukraine	1/2a	ST98	CC7
8	39	1956	guinea pig	Irkutsk region	1/2a	ST7	CC7
9	К-17	1956	rabbit	Ukraine	1/2a	**ST1546**	CC19
10	169	1957	louse	Ukraine	1/2a	**ST1534**	CC7
11	944	1958	house mouse	Moscow region	1/2a	ST7	CC7
12	2598	1960	rabbit	North Caucasus region	1/2a	ST7	CC7
13	97	1960	rabbit	Voronezh region	1/2a	**ST1550**	CC124
14	35	1962	sheep	Kazakhstan	1/2a	ST101	CC101
15	406	1964	pig	Kazan	1/2a	ST7	CC7
16	Chistopol	1964	pig	Volga region	1/2a	**ST1541**	CC7
17	121	1964	bovine	Moscow region	1/2a	ST124	CC124
18	3501	1965	goat	Moscow	1/2a	ST85	CC7
19	3453	1965	pig	Moscow	1/2a	ST7	CC7
20	324	1965	pig	Moscow	1/2a	ST23	CC7
21	27-T	1966	rat	Tajikistan	1/2a	ST7	CC7
22	3-P/2	1966	sheep	South Ural region	1/2a	ST7	CC7
23	2-P	1967	bovine	South Ural region	1/2a	ST177	CC177
24	119	1967	cow	Ural region	1/2a	**ST1542**	CC177
25	178-P	1967	pig	Uzbekistan	1/2a	**ST1535**	CC7
26	14P	1969	sheep	Altai region	1/2a	**ST1534**	CC7
27	3880	1970	pig	Ural region	1/2a	ST7	CC7
28	4-G	1970	sheep	Kazakhstan	1/2a	ST7	CC7
29	1426	1970	cow	Irkutsk region	1/2a	**ST1548**	CC101
30	1-CAX	1971	cow	Sakhalin region	1/2a	**ST1536**	CC7
31	45	1971	sheep	Kazakhstan	1/2a	**ST1459**	CC307
32	140	1971	cow	Belarus	1/2a	ST23	CC7
33	257	1971	cow	Novgorod region	1/2a	**ST1551**	CC124
34	24618	1971	human	Moscow	1/2a	ST7	CC7
35	57	1971	human	Moscow	1/2a	**ST1539**	CC7
36	174	1971	sheep	Belarus	1/2a	**ST1552**	CC124
37	50	1971	sheep	Belarus	1/2a	ST7	CC7
38	1-67	1972	sheep	Altai region	1/2a	**ST1537**	CC7
39	7-B	1972	sheep	Belarus	1/2a	ST481	CC18
40	170	1974	cow	Khabarovsk region	1/2a	ST177	CC177
41	33	1975	sheep	Chita region	1/2a	**ST1540**	CC7
42	305	1975	cow	Kazakhstan	1/2a	ST12	CC7
43	816-D	1975	goat	Dagestan region	1/2a	ST21	CC21
44	5 ch	1975	human	Tula region	1/2a	ST12	CC7
45	К-23	1988	human	Moscow	4b	ST252	CC1
46	211	1992	cow	Kursk region	1/2a	**ST1538**	CC7
47	76	1997	human	Tula	1/2a	**ST1547**	CC89
48	29 ch	1999	human	Tula region	1/2a	**ST1543**	CC177
